# Seventh Decennial Convention

**Published:** 1889-07

**Authors:** Robert Amory

**Affiliations:** President of the Convention of 1880; Boston


SEVENTH DECENNIAL CONVENTION for revising the Pharmacopœa of the United States of America.—Notice is hereby given that, in accordance with and by virtue of the authority vested in me by the convention of 1880, I hereby call upon the several incorporated Medical Societies, incorporated Medical Colleges, incorporated Colleges of Pharmacy, and incorporated Pharmaceutical Societies throughout the United States of America, the American Medical Association, and the American Pharmaceutical Association, to elect a number of delegates, not exceeding three, and upon the Surgeon-General of the Army, Surgeon-General of the Navy, and the Surgeon-General of the Marine Hospital Service to appoint, each, not exceeding three medical officers to attend a General Convention for the revision and publication of the Pharmacopœia of the United States of America, to assemble in the city of Washington, D. C., on the first Wednesday of May, 1890 (May 7th), at 12 o'clock noon.

The several bodies, as well as the Medical Departments of the Army, Navy and Marine Hospital Service, are hereby requested to submit the Pharmacopœia to a careful revision, and to transmit the result of their labors to the Committee of Revision at least three months before the meeting of the General Convention.

The several Medical and Pharmaceutical bodies are hereby requested to transmit to me, as the President of the Convention of 1880, the names and residences of their respective delegates, as soon as they shall have been appointed; a list of these delegates shall thereupon be published under my authority, for the information of the medical public, in the newspapers and medical journals in the month of March, 1890.

A blank form of certificate of appointment of delegates will be sent upon application by letter to my address, care of Dr. Edwin H. Brigham, Assistant Librarian of the Boston Medical Library, 16 Boylston Place, Boston, Mass.

(SIGNED) ROBERT AMORY,
Boston, March 8th, 1889. President of the Convention of 1880.

